# Heredity in Regard to Length of Life

**Published:** 1898-03-05

**Authors:** 


### HEREDITY IN REGARD TO LENGTH OF LIFE.

The great tendency displayed by modern clinical teachers to lay emphasis upon physical and demonstrable evidences of disease undoubtedly leads men to accept with far too great readiness the absence of such evidence of disease as proof of health.

This is a matter which is of great importance in regard to life insurance. The tendency of the inexperienced is to trust to what is visible and capable of investigation; but the medical advisers of the great insurance companies, who in many cases are able to trace long family histories in a way that is impossible even to that rapidly vanishing genus the old-fashioned family practitioner, know better, and, however satisfactorily a budding physician may report on a life, after the most complete investigation by aid of the most elaborate and modern instruments of precision, that life will not pass if the office finds that there is a bad family history. Hence, great interest attaches to the remarks made by Dr. Hermann Weber at a recent meeting of the Life Assurance Medical Officers' Association in regard to heredity in relation to life assurance.

He dealt with many diseases which need not concern us here, since if these diseases exist, or have existed, ordinary care will find out all about them. But he laid especial stress on conditions which are undiscoverable by physical research, namely, hereditary tendencies to certain groups of diseases and certain modes of degeneration, and, more particularly, the tendency to long life which is so manifest in some families, and the tendency to premature decay which is equally manifest in others. Putting on one side the majority of families, in which the average duration of life prevails—that is, in which those who reach adult age die between 60 and 72, unless some infectious disease or other unfavourable influence intervenes—he spoke (*a*) of long-lived, and (*b*) of short-lived families.

(*a*) The long-life tendency must not be regarded as consisting merely of a tendency to live on for a long time under ordinary circumstances. There seems to be in these families a greater vital resistance to evil influences, so that, even if accidents and illnesses happen, the patients make a better fight for life than people of a less fortunate and less hardy stock. This is a matter of great importance, because a careful recognition of the long-life tendency often enables insurance companies to accept lives with flaws which otherwise would be rejected. For example, if there is no other cause against it, gout inherited or acquired may in some such cases be accepted at ordinary rates. In certain cases of heart disease also it is possible to accept with a moderate addition to the premium cases which in average families could not be accepted at all, and the same is markedly the case in regard to phthisis, in which a recognition of the long-life tendency is a most important factor in the arriving at a correct estimation of the value of the life. A very interestin remark made by Dr. Weber is the following "A peculiarity occasionally observed in these long-lived families is, that not all the organs and functions retain their vigour in an equal degree, that old age shows itself occasionally even early in the hair, the teeth, the senses, the generative organs, and even in memory and the higher functions of the brain; but as these organs and functions are not vital, insurance officers need not mind very much."

(*b*) Short-lived tendencies are of even greater importance in life assurance than their opposites, for any failure to recognise the existence of this essential instability of constitution may lead to great loss. Dr. Weber says: "Although all offices recognise this tendency to early death, I venture to state that the disadvantages are not so generally recognised as they ought to be. I infer this from reports of country cases, and especially from the answers often given by the examiners to the question—is the family history good?" The short-lived tendency is not shown merely by a tendency to die at an early age from ill-defined conditions coming under the head of general breakdown, but by a lack of resisting power, a tendency to die from ordinary ailments through which other people pass without much danger.

The following case given by Dr. Weber is a striking instance of short-lived tendency showing itself in various members of a family: A man of 36, without occupation, without any organic disease, "healthy, but not vigorous," and "looking a little older." Father died at 51, pneumonia; mother at 48, change of life; 1 brother at 38, Bright's disease; 1 at 28, rheumatic fever; 1 died in infancy; 2 brothers living, 25 and 27; 1 sister died at 18, diphtheria; 1 at 24, diarrhœa; 1 at 32, in Australia, from exhaustion after sea voyage; 3 are living under 30, healthy. The history of uncles and aunts and of grandparents is not given. The answer to the ordinary question about good family history is "yes."

This life was recommended at ordinary rates, but, as is pointed out, the family history pointed to early death, and showed that the proposer was not an average life, notwithstanding the absence of obvious disease. It is a curious thing that, in these short-lived families, premature senility of one or several vital organs may be observed in successive generations. Thus, says Dr. Weber, "I sometimes see at present a man whose age is only 45, but who is in reality an old man in all his tissues and organs. He is grey, he has lost all his teeth, he is deaf, he has had to use glasses for more than six years, he has no memory, he has lost the control of the bladder, and he has been without sexual power for more than eight years. I have seen his father die under similar circumstances from general decay at the age of 49; and the late Dr. Addison, with whom I had attended the case, told me that exactly the same had happened with the grandfather. Insurance offices have to be very careful with such families. The man whom I have described just now has been insured at the age of 21 as an average life at two offices. With due attention to the family history, viz., to the tendency to premature decay and early death, this would not have happened." "Vitality" is a strange thing, of the essence of which we know nothing; but it is clear that in estimating a man's chances of long life the amount and the character of his "vitality" are quite as important points for consideration as the nature of the diseases from which he may have suffered, or the accidents to which he has been exposed.

